# Technology Adaptivity Mediates the Effect of Technology Biography on Internet Use Variability

**DOI:** 10.1093/geroni/igz054

**Published:** 2020-01-01

**Authors:** Alexander Seifert, Stefan T Kamin, Frieder R Lang

**Affiliations:** 1 Institute of Sociology, University of Zurich, Switzerland; 2 Institute of Psychogerontology, Friedrich-Alexander University of Erlangen-Nuremberg (FAU), Germany

**Keywords:** Attitudes, Biography, Internet use, Online participation, Technology

## Abstract

**Background and Objectives:**

Increasing numbers of older adults use the internet, but relatively little is known about the range and determinants of different online activities among older internet users. This study explores the interplay between technology-related biographical experiences and subjective technology adaptivity to explain the variability of internet use. Older adults who report having had more biographical experiences with technologies were expected to use a greater range of online activities. In addition, subjective technology adaptivity was expected to serve as a mediator of this relationship.

**Research Design and Methods:**

The analyses are based on a sample of 707 community-dwelling older participants of the University of the Third Age between 60 and 95 years of age (mean age = 72.49 years; 48% female) who use the internet. The measures include self-reports of online activities, technology-related biographical experiences, subjective technology adaptivity, and personal characteristics (age, gender, education, income, living-together status, and subjective health). Correlations and a path model with mediator effects were used to explore the research hypothesis.

**Results:**

The bivariate effects on the variability of internet use showed that study subjects participated in a greater range of online activities when they lived together with other people and were male, younger, and had higher levels of subjective technology adaptivity, technology-related biographical experiences, and educational level. The direct effects on the mediator show higher levels of subjective technology adaptivity for people who reported greater technology-related biographical experiences and for those who reported higher levels of subjective health.

**Discussion and Implications:**

The results show that the positive association between people’s past experiences with and stances toward technology in their own lifetimes and their range of diverse internet activities is mediated by subjective technology adaptivity. The findings also help to illustrate which biographical factors and which current individual factors explain differences in actual online behavior.

**Translational Significance**

This study examines how people’s past experiences with technology affect their current range of diverse internet activities. This association was found to be mediated by people’s subjective technology adaptivity. The study showed that people’s current online behavior is associated with their past and current experiences with and attitudes toward technology in general.

Internet use has become a key dimension of social and civic participation in everyday life in most modern societies and has also been shown to contribute to positive aging outcomes (e.g., Antonucci, Ajrouch, & Manalel, 2017; Cotten, Ford, Ford, & Hale, 2014; Kamin & Lang, 2018; Quintana, Cervantes, Sáez, & Isasi, 2018; Sims, Reed, & Carr, 2017). Although previous research has shown that older adults are less likely to use the internet than younger adults (e.g., Anderson & Perrin, 2017; Hunsaker & Hargittai, 2018; König, Seifert, & Doh, 2018), less is known about the variability and range of different online activities among older internet users as well as the determinants of such use. In this research, we have investigated the interplay between *technology-related biographical experiences* and *subjective technology adaptivity* to explain the individual ranges of internet activities among older internet users. “Biographical experiences” refer to one’s previous experiences and stances toward technological environments accumulated throughout one’s life (Mollenkopf & Kaspar, 2004); whereas “subjective technology adaptivity” reflects one’s current motivational resources to use technology in daily life (Kamin & Lang, 2013).

We expected that older adults who report more biographical experiences—thus indicating a stronger innovation orientation and technical interest throughout life—would use a greater range of internet activities. We also expected that subjective technology adaptivity could serve as a mediator of this relationship.

## Theoretical Assumptions

The construct of technology biography is based on the concept of *technology generations* (Sackmann & Winkler, 2013). According to this concept, members of different age cohorts experience different types of technology-related socialization in their youth. Although younger adults—members of what Sackmann and Winkler (2013) call the “internet generation”—are more familiar with different online activities such as social media, blogging, and online banking, older internet users often report more restricted and basic usage behaviors (Pew Research Center, 2018; Seifert & Rössel, 2019). For example, Büchi, Just, and Latzer (2016) found that age is associated with less frequent, and more narrow, uses of internet resources. In this vein, Seifert and Schelling (2016) reported that the older adults included in their study used the internet more for activities such as emailing, information seeking, and travel planning and less for social networking and entertainment.

Earlier research has shown, however, that variability exists both between different age cohorts and within age cohorts (Dannefer, 1987). For instance, statistics show that internet use is still characterized by a digital divide between age and cohort groups (Hunsaker & Hargittai, 2018). Some researchers have also observed that older adults who report having experience with computers in their work lives are more likely to use the internet after retirement (Chang, McAllister, & McCaslin, 2015; König, Seifert, & Doh, 2018). A few studies have also reported an association between previous computer experience and online proficiency among older adults (Boot et al., 2015). Although such findings primarily include binary information of internet use (i.e., people either do or do not use the internet), the use of more comprehensive measures of online participation involving both the frequency and the variability of different online activities (Hunsaker & Hargittai, 2018) is often recommended in current research.

Based on the unified theory of acceptance and use of technology (UTAU), work-related experience with technological innovations may be an important moderator for behavior intention regarding the acceptance of new technologies because people’s current intention is typically built on their acquired skills and evaluations of previous technologies (Venkatesh, Morris, Davis, & Davis, 2003). In addition to online internet activities during old age being associated with the acceptance of internet usage in general, we argue that such activities might also be associated with the previous experiences older people have had throughout their lives. This situation would mean that older people who have previously shown interest in and familiarity with technological innovations might be better able to embrace a richer, more differentiated online experience today. Thus, we submit that people who have experienced richer and more diverse technological environments are more likely to build motivational resources related to technology, which, in turn, will influence their actual online usage behavior.

In the current study, we propose that *subjective technology adaptivity* reflects such a *personal motivational resource*, which refers to a sense of competence when dealing with technological innovation (Kamin & Lang, 2013). This motivational resource explains how older people evaluate their current technological environments in relation to three aspects of motivation: (a) *perceived adaptive utility* refers to people’s beliefs that technology helps them to exert control and autonomy in their everyday lives; (b) *technology-related goal engagement* reflects the capacity to invest one’s behavioral resources and efforts when using technology; and (c) *perceived safety of technology* relates to feelings of dependability, trust, and safety while interacting with technological products. These dimensions are conceptually different, but their covariation indicates the common factor of subjective technology adaptivity. Cross-sectional and longitudinal findings have shown that subjective technology adaptivity predicts technology use among older adults (Kamin, Lang, & Beyer, 2017), but whether this construct will explain the variability in internet use remains unknown.

In addition to the direct association between technology experiences and variability of internet use, we argue that a meditation effect also exists with subjective technology adaptivity. This interplay may help us to better understand why previous experiences with technology are associated with the variability of internet activities. At least to a certain extent, subjective technology adaptivity might be determined by one’s previous experiences and stances toward technology. For example, people who have had positive experiences with computers in the workplace might be more likely to have positive views of technological innovations in general, which may also contribute to their perceived competence and adaptivity in response to technological change. This assumption is theoretically consistent with personality research in suggesting that the motivational processes of a person’s personality are shaped by their social and contextual influences (Costa & McCrae, 1997; McAdams & Pals, 2006).

### Research Aim

This research investigates the association between technology-related biographical experiences and subjective technology adaptivity to explain the variability of internet activities among older internet users. Given the arguments noted above, we hypothesize that subjective technology adaptivity acts as a mediator in the relationship between technology-related biographical experiences and the variability of internet activities.

## Method

### Participants

The study is based on data drawn from a representative survey (Seifert, 2019) of 811 participants at the University of the Third Age at the University of Zurich and ETH Zurich, the Swiss Federal Institute of Technology, Switzerland (UZH3). The study was a self-guided survey and was administered either via paper and pencil or alternatively, online. All members of the UZH3 were invited, through a posted invitation, to participate in the study and no financial incentives were offered. The response rate of this survey was 28%. UZH3 offers periodic open lectures from different departments on various scientific topics for an annual participation fee. The survey participants had attended a talk an average of 12 times during the previous 12 months (standard deviation [*SD*]: 10.89). The participants included in this study were at least 60 years old, used the internet, and provided full information on the variables of interest. The sample included 707 participants, with an average age of 72.49 years (*SD* = 5.97); 48% were female. Table 1 provides a description of the sample.

**Table 1.** Descriptive Statistics and Correlations (*N* = 707)

	*M* (*SD*) or %	Range	Correlations									
			1	2	3	4	5	6	7	8	9	10
1. Age	72.49 (5.97)	60–95	−									
2. Female	48%	–	−.10*	–								
3. Education	4.36 (1.23)	1–6	−.07	−.23***	–							
4. Household income	4.11 (1.26)	1–6	−.11**	−.31***	.25***	–						
5. Living together	65%	–	−.12**	−.38***	.09*	.50***	–					
6. Perceived health	5.27 (.52)	3–6	−.26***	.05	−.01	.18***	.09*	–				
7. Retired	89%	–	.29***	−.07	.00	−.09*	−.07	−.05	–			
8. Frequency of internet use	2.60 (.41)	1.14–3.93	−.15***	−.01	.12**	.09*	−.03	.14***	−.09*	–		
9. Tech biography	3.62 (.90)	1–5	−.07	−.39***	.15***	.17***	.13***	.13***	−.04	.21***	–	
10. Technology adaptivity	3.54 (.67)	1.33–5	−.06	−.14***	.06	.10*	.05	.21***	−.06	.38***	.57***	–
11. Variability of internet use	12.65 (2.91)	2–17	−.35***	−.23***	.18***	.22***	.25***	.14***	−.16***	.21***	.43***	.46***

*Notes:* 1. Age (age in years); 2. Female (ref. male); 3. Education (education level from 1 “pre-primary education” to 6 “second state of tertiary education”); 4. Household income (monthly income of the household in Swiss francs [CHF]); 5. Living together (household composition, living together [ref. living alone]); 6. Perceived health (self-rated health on a 6-point scale from 1 “poor” to 6 “very good”); 7. Retired (ref. not retired); 8. Frequency of internet use (calculated as the mean across the 17 possible internet activities, scaled from 1 “less than once a month” to 4 “daily”); 9. Tech biography (Technology biography scale, measured on a 5-point scale); 10. Technology adaptivity (Subjective Technology Adaptivity Inventory, measured in a 5-point scale); 11. Variability of internet use (counting score of 17 different internet activities).

*p < .05.

**p < .01.

***p < .001.

### Measures

Variability of internet use was assessed with a list of 17 different internet activities (e.g., chatting, streaming, online shopping, information seeking, social media usage, and online banking). Participants indicated whether they had engaged in the respective activity over the previous 3 months. We calculated scores by counting the activities to indicate the diversity of internet usage behavior (Seifert & Schelling, 2016).

Technology biography was measured with seven items, which participants indicated their agreement with on a 5-point scale (1 = *does not apply* to 5 = *fully applies*). Examples of the items included “I’ve always had a lot to do with technology in my life,” “A job related to technology would not have been for me” (reverse coded), and “I’ve always been interested in learning how to handle new devices.” These items, which were drawn from previous research by Mollenkopf and Kaspar (2004), reflect biographically related experiences and stances toward technological environments in an older person’s past. We calculated the mean of all items, with higher values indicating a stronger innovation orientation and technical interest throughout life, whereas lower levels indicated past technology avoidance and distance. The Cronbach’s alpha of the scale was .87.

Subjective technology adaptivity was assessed with the Subjective Technology Adaptivity Inventory (Kamin & Lang, 2013; Kamin et al., 2017). We used a short version with 9 items. Participants answered the items on a 5-point scale (1 = *do not agree* to 5 = *absolutely agree*). Examples of items included “I put in more effort when a new device is more difficult to use than expected,” “I believe that modern technology conforms to safety standards,” and “Using technology helps me to be more efficient in my daily routines.” We calculated the mean of all items, with higher scores reflecting a greater extent of subjective technology adaptivity. The Cronbach’s alpha for the scale was .88.

Covariates included chronological age in years, sex (0 = male; 1 = female), educational level (1 = preprimary education to 6 = second state of tertiary education), monthly household income (1 = up to 2,000 CHF [Swiss francs] to 6 = over 10,000 CHF), retirement (0 = not retired; 1 = retired) and household composition (0 = living alone; 1 = living together). In addition, we included a scale reflecting perceived health based on 6 items measured on a 6-point scale (1 = *poor* to 6 = *very good*). The items were adapted from the Short-Form Health Survey SF-36 (Ware & Gandek, 1998) and included different health-related domains, such as quality of life, health, memory, mobility, and daily functioning. Finally, we included the frequency of internet use as an additional covariate to control for differences between the variability and the frequency of use (e.g., people who excessively use the internet for only a few specific activities might show lower usage variability and vice versa). The frequency of use was calculated as the mean across the 17 internet activities (1 = [*less than once a month*] to 4 [*daily*]).

### Analytical Strategy

**Figure 1.**
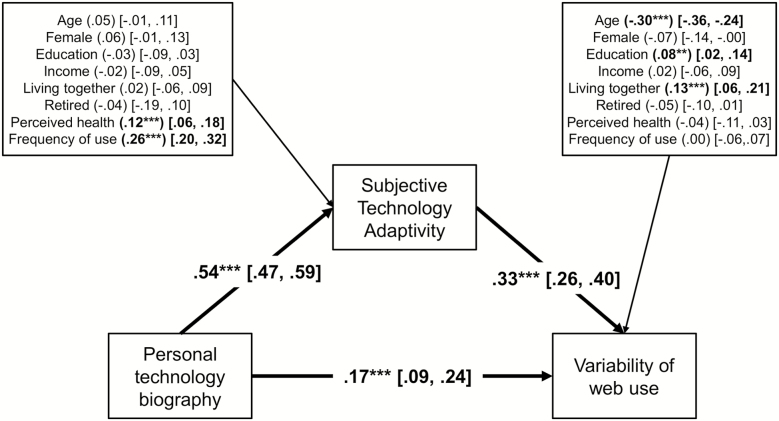
Mediation model; standardized estimates; coefficients in brackets indicate 95% confidence intervals; bold faced coefficients indicate significance; *p < .05; **p < .01; ***p < .001.

We fitted a path model (Figure 1), with the variability of internet use as a dependent variable, on technology-related biographical experiences, subjective technology adaptivity, and covariates (age, gender, education, income, perceived health, and frequency of use). Subjective technology adaptivity was included as a mediating variable and controlled for all covariates. Using this approach allowed us to fit a single model while simultaneously controlling for covariates, rather than estimating different regression models (Hayes, 2013). Bootstrapping with 5,000 draws was used to calculate standard errors for the total, direct, and indirect effects. All analyses were conducted using Stata 15 software.

## Results

Table 1 shows the descriptive statistics and correlations. Variability of internet use was positively associated in a bivariate correlation with education, income, subjective health, technology-related biographical experiences, subjective technology adaptivity, and the frequency of internet use. In addition, in this study, the older participants and those who were retired were more likely to show less variability in their internet activities compared to younger participants.

Figure 1 shows the estimated mediation model. The direct effects on the variability of internet use showed that people participated in a greater range of online activities when they lived together with other people and had higher levels of subjective technology adaptivity, technology-related biographical experiences, and educational level. The variability of internet use was lower among older people. The direct effects on the mediator showed higher levels of subjective technology adaptivity for people who reported more technology-related biographical experiences and for those who reported higher levels of subjective health. In addition, the frequency of Internet use was associated with higher levels of subjective technology adaptivity. The effect of biographical experiences without the mediator was .34 (*p* < .001). When including subjective technology adaptivity as a mediator, the effect of biographical experiences was reduced to .17 (*p* < .001), indicating the indirect effect of this variable through subjective technology adaptivity. The respective standardized indirect effect was .17 (*p* < .001), which suggests that subjective technology adaptivity accounted for 50% of the total effect of biographical experiences on the variability of internet use.

We performed a supplementary analysis to address the methodologic concern that technology biography and subjective technology adaptivity may reflect overlapping constructs. Findings from an exploratory factor analysis provided evidence that technology biography and subjective technology adaptivity are distinct constructs (Supplementary Table 1).

## Discussion

This is the first study to have explored the effect of the interplay between technology-related biographical experiences and subjective technology adaptivity on individual variability in utilizing different online activities among a large representative sample of older internet users. Our findings provide evidence for a relationship between people’s past experiences with technology and their current evaluation of technology in terms of the diversity of their online participation. The study has shown that subjective technology adaptivity accounted for half the total effect of biographical experiences on the variability of internet uses. These findings add to the literature in three ways, as follows.

First, we were able to show that subjective technology adaptivity mediated the effect of biographical experiences on the variability of internet use. That is, older adults who reported more biographical experiences with technology were more likely to use a greater variety of internet activities because they also reported higher levels of subjective technology adaptivity. This finding illustrates how people’s previous experiences within technological environments interact with their motivational resources when using technology. Although researchers have argued that people’s previous experiences with technology account for differential usage behaviors in later life (Fozard & Wahl, 2012; Sackmann & Winkler, 2013), the field’s knowledge about the psychological mechanisms underlying this association remains limited. This study is the first to have indicated a possible pathway in which previous experiences with technology affect the variability of online activities through individual differences in subjective technology adaptivity. Such findings help to explain the variability and diversity of digital innovation among older adults. For example, individuals’ biographical experiences may not be strongly associated with the variability of internet use when they lack motivational resources related to the actual use of technology. Moreover, biographical experiences may affect the use of digital innovations indirectly through technology-related motivational resources, even if there is no direct association with usage behavior. For example, being able to learn how to use a new device may build on the fundamentals of prior experience in technological environments; but the motivational capacity to invest effort and time in overcoming usability obstacles may explain for the relation between experience and usage behavior.

Second, our study shows the ongoing relevance of subjective technology adaptivity and technology-related biographical experiences for explaining the use of technology among older adults (Czaja et al., 2006; Kamin & Lang, 2013; Kamin et al., 2017). We were further able to show the relevance of both factors for the variability of active online use, which van Deursen and van Dijk (2014) call the “second-level divide,” and not only for the first level of the digital divide which refers to having basic internet access. Therefore, our findings contribute to an improved understanding of which interindividual differences lead older internet users to grasp the various potentials the internet has to offer.

Third, we found that age was significantly associated with the variability of internet use but not with subjective technology adaptivity. This noteworthy finding suggests that people’s technology-related motivational resources might be less dependent on chronological age but may rather be conditional on their socialization experiences throughout life. Consequently, being older may not necessarily influence people’s motivational resources related to their use of technological innovation (Berkowsky, Sharit, & Czaja, 2017); rather, their formative experiences within technological environments may affect their motivational capacity to adapt to technological changes later in life.

Despite the strengths of this study, several limitations must be noted. First, the present study had a specific regional focus, so our findings have limited generalizability. Second, one could argue that the sample of active participants at the University of the Third Age is selectively biased. Nevertheless, we think that this selected group represents a heterogenous group of older internet users in Switzerland. Third, the mediation model provided only a cross-sectional view of the data. It is not possible to draw definitve conclusions about the causal ordering of the variables examined. Moreover, we cannot rule out alternative directions of associations. For example, it is possible that individuals with higher motivational resources are more likely to seek out biographically relevant technological environments, which in turn, may affect their motivation. Clearly, future research should investigate the dynamics of those interplays against the background of today’s persistent digital transformation. Fourth, because of the limited width of the study variables we used, we were unable to control for other important background factors, such as technophobia, personality, internet skills, or attitudes toward the internet. Further studies using longitudinal designs and with a wider range of variables will therefore be required to examine this topic in more detail.

The following practical recommendations have emerged from the present study. Based on our findings, the intraindividual flexibility of online participation appears to be influenced both by people’s previous experiences with and stances toward technology and by their current motivational resources regarding technologies in general. For this reason, the use of educational interventions that target motivational aspects of internet use, in addition to the important level of internet skills (Hargittai, Piper, & Morris, 2019), could enrich people’s openness to new online activities. Such interventions might include a presentation of the variety and possibilities of online activities, which could then contribute to older people’s experience of control and independence (Quan-Haase, Williams, Kicevski, Elueze, & Wellman, 2018; Shapira, Barak, & Gal, 2007). In addition, our findings point to the possible relevance of biographical work with older adults. Exploring life histories may help to understand the circumstances, experiences, opportunities, and obstacles that have shaped their experience of technology. Consequently, improved understanding of a person’s biography may serve to identify possible strategies for stimulating and activating his or her motivational resources of technology usage (e.g., by remembering situations where new technology was interesting and useful). Furthermore, the special learning needs of older adults also need to be considered in the design of technologies (Cotten, Yost, Berkowsky, Winstead, & Anderson, 2016;Czaja, Boot, Charness, & Rogers, 2019).

## Supplementary Material

Supplementary data are available at *Innovation in Aging* online.

Supplementary Table 1. Factor loadings of the technology biography scale and the subjective technology adaptivity subscales (*N* = 728)

igz054_suppl_Supplementary-Material

## Conflict of Interest

None reported.
