# Supplementary material for: Technology Adaptivity Mediates the Effect of Technology Biography on Internet Use Variability
Source: Innov Aging. 2020 Jan 1;4(2):igz054. doi: 10.1093/geroni/igz054 (PMC6938462; doi:10.1093/geroni/igz054)
Supplement: igz054_suppl_Supplementary-Material [file igz054_suppl_supplementary-material.docx]

Supplementary Table 1. *Factor loadings of the technology biography scale and the subjective technology adaptivity subscales (N = 728)*

| Items | Technology biography (TB) | Perceived adaptive utility (PAU) | Technology-related goal engagement (TGE) | Perceived safety of technology (PST) |
| --- | --- | --- | --- | --- |
| TB1 | **.81** | .06 | -.09 | -.03 |
| TB2 | **.84** | -.05 | -.14 | -.01 |
| TB3 | **.80** | -.03 | -.02 | -.01 |
| TB4 | **.52** | .11 | .08 | .12 |
| TB5 | **.62** | -.03 | .11 | -.04 |
| TB6 | **.43** | .01 | .29 | .02 |
| TB7 | **.50** | -.00 | .35 | .01 |
| PAU1 | -.02 | **.79** | .10 | -.07 |
| PAU2 | -.03 | **.80** | -.03 | .10 |
| PAU3 | .05 | **.80** | .02 | -.00 |
| TGE1 | -.04 | .03 | **.84** | -.01 |
| TGE2 | -.07 | .03 | **.97** | -.04 |
| TGE3 | .02 | .01 | **.75** | .10 |
| PST1 | .06 | .02 | .10 | **.66** |
| PST2 | -.05 | -.08 | -.03 | **.86** |
| PST3 | -.02 | .14 | -.01 | **.75** |

*Note.* Factor solutions were obtained by exploratory factor analysis with maximum likelihood estimation and promax rotation.
